# Bone Resorption Assessment Following Zygomatic Implants Surgery over 10 Years of Follow-Up

**DOI:** 10.3390/jcm14030989

**Published:** 2025-02-04

**Authors:** Fernando Duarte, Carina Ramos, Juan Santos-Marino, Natalia Martínez-Rodriguez, Cristina Barona-Dorado, José María Martínez-González

**Affiliations:** 1 Department of Surgery, Faculty of Medicine, University of Salamanca, 37007 Salamanca, Spain; fduarte@clitrofa.com (F.D.); juansantos@usal.es (J.S.-M.); 2 Clitrofa Education Academy, 4785-248 Trofa, Portugal; cramos@clitrofa.com; 3 Department of Clinical Dental Specialities, Complutense University of Madrid, 28040 Madrid, Spain; cbarona@ucm.es (C.B.-D.); jmargo@ucm.es (J.M.M.-G.); 4 Surgical and Implant Therapies in the Oral Cavity Research Group, Complutense University, 28040 Madrid, Spain

**Keywords:** zygomatic implant, zygoma bone resorption, long-term bone resorption

## Abstract

The presence of sufficient bone volumes is one of the most important criteria for the success of oral implant osseointegration. Therefore, the rehabilitation of edentulous atrophic maxillae represents the greatest challenge in terms of oral rehabilitation. Techniques such as bone grafts, angled implants, short implants, tuberosity, and pterygoid implants may not always be a viable alternative in the subsequent rehabilitation of the posterior atrophic maxilla. A breakthrough occurred when Brånemark first introduced longer, custom-designed implants inserted into the zygomatic bone to support craniofacial prosthesis in the 1980s. When used in the treatment of atrophic jaws, zygomatic implants provide a safe and effective alternative, with stable long-term results. **Objectives**: We aimed to retrospectively evaluate zygomatic bone resorption ten years after the placement of zygomatic implants. **Methods**: A retrospective observational study was designed to evaluate bone resorption over ten years following the placement of zygomatic implants. In a study group of 50 patients, using Hounsfield scales, the area of the zygoma and its bone density were established and evaluated. The NewTom NNT Analysis software (NewTom^®^, Imola, Italy) was employed to trace the bone and implant limits on CBCT scans. Using this software, the three-dimensional information of the postoperative CBCT image was compared with the ten-year postoperative CBCT image, allowing for the assessment of the zygomatic bone resorption and bone density. **Results**: Highly significant statistical differences to an alpha level of 0.01 were identified between T0 (pre-op), T1 (12 months), and T2 (120 months) concerning zygomatic bone density, both in the first and in the second quadrants. The post hoc Bonferroni test revealed that significant statistical differences were observed between T0 and the remaining timepoints (T1 and T2), with the latter two exhibiting similar values. **Conclusions**: The evaluation of the resorption at the level of the zygoma, ten years after the placement of zygomatic implants, reveals that there are no significant losses between the initial and final controls. Therefore, it follows that this type of implant rehabilitation represents a viable alternative approach in patients with bone atrophy of the maxilla, offering a predictable therapeutic solution that enables immediate full function and excellent long-term success rates. However, we must not neglect the potential for future innovations in GBR involving the use of barrier membranes, either resorbable or non-resorbable, and even the application of titanium alveolar customized osteogenic scaffold, in conjunction with autologous bone grafts or biomaterials.

## 1. Introduction

Maxillary edentulism is increasingly prevalent globally. The World Health Organization (WHO) indicates that the primary etiological factors for tooth loss include a chronic history of oral diseases, notably extensive dental caries, and advanced periodontal disease. Additionally, tooth loss may arise from other factors such as traumatic injuries, pathological conditions, infections, and various systemic health issues [1]. Cardiovascular conditions and bleeding disorders that interfere with invasive procedures can affect surgery, healing, and implant maintenance. Systemic and local factors may also compromise bone and soft tissue healing after implant insertion [2]. Important systemic factors include osteoporosis, anti-bone resorptive medications, diabetes mellitus, and immune deficiency, and behavioral factors include substance abuse, in particular tobacco and alcohol. Local factors that should be considered include history of radiation therapy or oral mucosal diseases [2].

Over the last 30 years, the number of individuals with edentulism has doubled. A decrease in the incidence rate of edentulism was found in younger age groups, and there was a shift in the peak age of prevalence from 70 to 74 in 1990 to 75–79 in 2021 [3]. Globally, females have a higher prevalence rate of edentulism than males, with a disproportionate burden in regions with a high demographic index [3].

Edentulism remains a significant global public health concern, with the global prevalence rate projected to increase to 5004 individuals per 100,000 in 2040 [3]. The loss of teeth can have various implications, including psychological challenges, social impacts, and functional limitations. The effects of edentulism may diminish an individual’s ability to consume food, leading to potential difficulties in chewing. This situation can result in notable changes in nutrition and may increase the risk of conditions such as obesity, diabetes, coronary artery disease, and certain types of cancer. Addressing these issues with care and attention is essential for overall well-being [1].

As the global population ages, tooth loss among older adults has become increasingly common, impacting oral function and overall health. Although removable dentures have been used to improve masticatory function, the long-term effects of denture conditions on mortality remain unclear [4]. After tooth loss, resorption of the alveolar bone in the maxilla occurs in a posterior/superior and lateral-to-medial direction [1]. Tooth loss is associated with the accelerated progression of frailty, and utilizing dentures did not reduce this trend [5].

The process of sinus pneumatization, combined with the resorption of alveolar bone, can potentially lead to a reduction in both vertical and horizontal bone volume in the posterior region of the jaw. Furthermore, a lack of significant anterior alveolar bone resorption may pose challenges to the use of conventional implants. It is also worth noting that the extended use of complete dentures may contribute to an increased degree of maxillary atrophy [6,7].

Alternative treatments are available for the atrophic maxilla, some of which use bone grafts, such as the iliac crest block bone graft, Le Fort I osteotomy, onlay-type bone grafting techniques, or maxillary sinus lift procedures in the posterior sectors of the maxilla [8]. Bone augmentation techniques, including guided bone regeneration (GBR), have emerged as indispensable tools for the treatment of complex defects. GBR involves the use of barrier membranes, resorbable or not, in conjunction with autologous bone grafts or biomaterials. The selection of membrane type and graft material depends on the size, shape, and complexity of the defect [9]. These techniques have several disadvantages: the need for multiple surgeries; the use of extraoral donor areas, which involves extra morbidity; and the length of time the patient has to spend without oral rehabilitation waiting for graft consolidation and healing. An alternative solution is to place implants in the zygomatic bone [8].

Zygoma implants were first introduced by Brånemark in 1988 with the original purpose of rehabilitating patients who had undergone maxillectomy due to tumor resection, trauma, or congenital defects [10,11,12]. However, the function of this implant has since expanded to the rehabilitation of patients with severely resorbed edentulous maxilla [10,12,13].

The original zygomatic Brånemark protocol included one implant on each zygoma, traversing the sinus, and splinted to 2 to 4 conventional implants in the anterior region [7]. Since then, numerous modifications to zygomatic implant designs, surgical approaches, and loading protocols have been documented in the literature with quite favorable results [12,14].

However, the survival of these implants has been assessed based on possible complications and the resorption that occurs at the crest of the maxilla [15,16]. There are no studies in the literature that evaluate the resorption that may occur in the main anchorage bone, which is the zygomatic bone. Therefore, the objective of this research is to retrospectively analyze ten years of rehabilitation with zygomatic implants, assessing whether there are losses at the level of the zygoma, proposing as a null hypothesis that these implants remain stable over time without the presence of bone resorption at the level of the zygomatic bone.

## 2. Materials and Methods

### 2.1. Research Design

A retrospective observational study was designed to evaluate patients treated with zygomatic implants. The participants in this study came from the Master of Science in Oral Surgery and Implantology of the Faculty of Dentistry, Universidad Complutense Madrid. This study was conducted in accordance with the provisions laid down in the Declaration of Helsinki. The protocol was submitted and approved by the CEIM Hospital Clínico San Carlos ethics committee with the internal identification number 24/522-E.

### 2.2. Sample

The clinical records of patients rehabilitated with two zygomatic implants and with a follow-up of at least ten years were reviewed. The inclusion criteria were as follows: (1) total edentulism of the maxilla; (2) performance of CBCT studies (NewTom 5G^®^, Imola, Italy) prior to surgical treatment, one year after, and ten years after prosthetic loading; and (3) acceptance of patients for their participation and scientific dissemination of their results. The exclusion criteria were as follows: (1) patients with severe osteoporosis; (2) patients who during the ten-year period have been treated pharmacologically with corticosteroids, antiresorptives, or chemotherapy; (3) patients who did not comply with the follow-up protocol; and (4) patients who did not give their consent for the use of their data.

After applying these criteria, a total of 50 patients were selected and rehabilitated following the principles of the ZAGA technique.

The implants were placed according to the principles of the ZAGA technique, the acronym for the Zygoma Anatomy-Guided Approach, a concept used to place zygomatic implants in a prosthetically driven manner and according to the anatomy of the patient. The ZAGA method aims to promote patient-specific therapy by adapting the osteotomy type and implant design to the patient’s structure. This acknowledges the existence of inter-individual anatomical differences as well as intra-individual variations. In most cases, this approach avoids the opening of a window or slot into the lateral wall of the maxillary sinus before implant placement. Instead, a mucoperiosteal flap, including the posterior maxillary wall and the superior zygomatic rim, is raised, to allow for visual control of the complete surgical field [17].

After one year and ten years, new follow-up scans were obtained. With the NewTom NNT Analysis software, version 16.4 (NewTom^®^, Imola, Italy), we were able to trace the bone and implants on CBCT scans. This software allowed for the three-dimensional information of the postoperative CBCT image to be compared with the ten-year postoperative CBCT image and the assessment of zygomatic bone resorption and bone density.

The following parameters were used: X-ray source—110 KV, 1–20 mA (pulsed mode); focal spot—0.3 mm; acquisition technique: single scan; scan time 18–36 s’ exposure; X-ray emission time 3.6 s–6.7 s; signal gray-scale—14-bit scanning and 16-bit reconstruction; FOV size DxH—6 × 6 cm; patient positioning—supine.

The zygomatic implant model used was manufactured by S.I.N.—Implant System (São Paulo, Brazil), with the following specifications: implant structure: apical diameter of Ø4.0 mm, cervical diameter of Ø4.4 mm, body diameter of Ø4.0–Ø4.4 mm. The available lengths were as follows: 32, 35, 37, 40, 42, 45, 47, 50, 52, 55, 57, 60, and 62 mm, and a mechanized surface with double acid etching. Prosthetic platform: universal external hexagon connection, 45° angled head, made from titanium grade IV.

### 2.3. Data Collection Instruments

This evaluation was performed before surgery (T0), 12 months after surgery (T1), and 120 months after surgery (T2), according to the previously described protocol.

**Figure 1:**
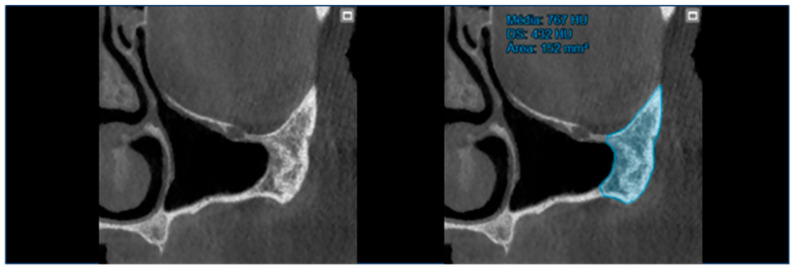
T0 preoperative assessment of zygomatic bone area and density.

**Figure 2:**
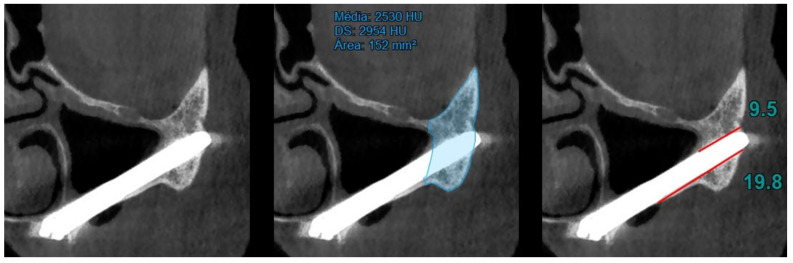
T1 evaluation considering (I) area and density of the zygomatic bone, (II) upper contact distance between the zygomatic bone and the zygomatic implant, and (III) lower contact distance between the zygomatic bone and the zygomatic implant.

**Figure 3:**
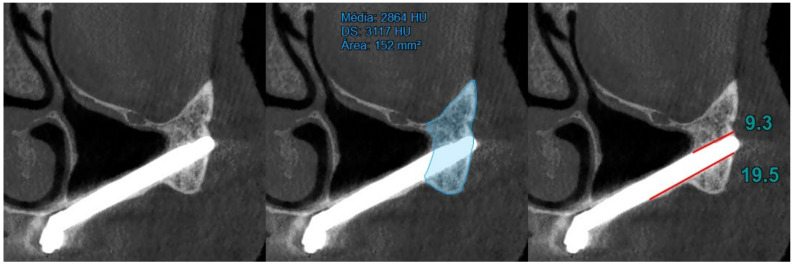
T2 evaluation considering (I) area and density of the zygomatic bone, (II) upper contact distance between the zygomatic bone and the zygomatic implant, and (III) lower contact distance between the zygomatic bone and the zygomatic implant.

- At T0 (preoperative), the area and bone density of the zygomatic bone were recorded (Figure 1).
- At T1 (12 months of follow-up), the area and density of the zygomatic bone, the upper contact distance between the zygomatic bone and the zygomatic implant, and the lower contact distance between the zygomatic bone and the zygomatic implant were evaluated (Figure 2).
- At T2 (120 months of follow-up), the area and density of the zygomatic bone, the upper contact distance between the zygomatic bone and the zygomatic implant, and the lower contact distance between the zygomatic bone and the zygomatic implant were also evaluated (Figure 3).

### 2.4. Statistical Analysis

IBM^®^ SPSS^®^ statistics software, version 30, was used for both descriptive and inferential data analysis. First, the study variables were tested to ensure that they conformed to a normal distribution by using either the Kolmogorov–Smirnov (D) or the Shapiro–Wilk (W) test. Secondly, the variances of the samples were tested for homogeneity by using either Levene’s (L) or Bartlett’s (B) test.

Descriptive statistics for the study variables included the calculation of the arithmetic mean ($\bar{x}$) and standard deviation (SD) for continuous variables, median and inter-quartile range (IQR) for ordinal variables, and mode and frequencies for nominal variables.

Inferential statistics were used for the comparison of the three experimental timepoints (T0, T1, and T2). Given the paired nature of the data, and once the requirements for normality of distribution and homogeneity of variance were confirmed, a repeated-measures ANOVA test was applied. The post hoc Bonferroni inferential test was used for the testing of multiple comparisons in situations where a statistically significant difference was identified by the repeated-measures ANOVA.

*p*-values below the statistical significance level (α) of 0.05 were considered statistically significant (*), below 0.01 as highly statistically significant (**), and below 0.001 as very highly statistically significant (***).

## 3. Results

Table 1 shows the sociodemographic and general clinical characterization of the 50 patients included in this study. The mean age was 60.32 ± 10.43, with a higher percentage of female patients (66%) compared to male patients (34%).

Regarding ZAGA characterization, in the first quadrant, ZAGA I was applied in 80% of cases, followed by ZAGA II in 12% and ZAGA 0 in 6% of cases. In the second quadrant, ZAGA I was applied in 74% of cases, followed by ZAGA II in 16% and ZAGA 0 in 10% of cases.

The most used zygomatic implant length in the first quadrant was 35 mm in 24% of the cases and in the second quadrant was 37.5 mm in 28% of the cases. The mean zygomatic implant torque in the first quadrant was 48.70 ± 11.19 N, and in the second quadrant, it was 49.70 ± 10.95 N.

Regarding the loading protocol, in 80% of cases, immediate function was performed, in 14% it was delayed, and in 6% it was early. With regard to antagonist occlusion, 52% of cases were total fixed implants rehabilitation, 30% natural teeth, 8% overdenture, 8% partial fixed implants rehabilitation, and 2% removable partial denture.

In this study, 8% of complications were recorded, present in four clinical cases, among which the following stand out: loss of a standard implant in anatomical position 2.1, two cases of sinusitis postop, and a fracture of the second quadrant zygomatic implant.

Table 2 presents the specific clinical characterization of the sample concerning the standard implants placed in the anterior maxilla. Implant 1 was mainly placed in anatomical position 1.1, in 54% of the cases, with the most used length being 3.75 × 10 mm, in 24%, and the mean torque obtained was 43.10 ± 11.51 N. Implant 2 was predominantly placed in anatomical position 1.3, in 36% of the cases, with the most used length being 3.75 × 10 mm, in 30%, and the mean torque obtained was 40.30 ± 13.49 N. Implant 3 was most frequently placed in anatomical position 2.1, in 38% of the cases, with the most used length being 3.75 × 8.5 mm, in 14%, and the mean torque obtained was 43.68 ± 14.17 N. Finally, implant 4 was placed in most cases in anatomical position 2.3, in 28% of the cases, with the most used length being 3.75 × 10 mm, in 22%, and the mean torque obtained was 37.68 ± 16.70 N.

Table 3 represents the patients’ follow-ups (T0, T1, and T2), concerning (i) zygomatic bone area (first and second quadrants); (ii) zygomatic bone density (first and second quadrants); (iii) upper contact distance between zygomatic bone and zygomatic implant (first and second quadrants); and (iv) lower contact distance between zygomatic bone and zygomatic implant (first and second quadrants).

Table 4 shows the results of the repeated-measures ANOVA (F) inferential test for comparing T0, T1, and T2 of the patients’ follow-ups (T0, T1, and T2), concerning: (i) zygomatic bone area (first and second quadrants); (ii) zygomatic bone density (first and second quadrants); (iii) upper contact distance between zygomatic bone and zygomatic implant (first and second quadrants); and (iv) lower contact distance between zygomatic bone and zygomatic implant (first and second quadrants). *p*-values below the statistical significance level (α) of 0.001 as very highly statistically significant (***).

In Table 5, the post hoc Bonferroni inferential test for comparing T0, T1, and T2 of the patients’ follow-ups (T0, T1, and T2) concerning zygomatic bone density (first and second quadrants) is shown. *p*-values below the statistical significance level (α) of 0.05 were considered statistically significant (*).

## 4. Discussion

### 4.1. Implant Survival

In Brennand’s review, zygomatic implant survival was 96.2% during a mean follow-up of 75.4 months (6.3 years) [18]. A higher incidence of failure was identified in the first year (2%) compared to subsequent years (0.5%/year), while the overall annual incidence of failure was 0.7%, with mean intra-study follow-up times ranging from 36 to 142 months. The results of this study align with the values reported in the systematic reviews presented by Chrcanovic [19,20]. These suggest that some patients may experience early postsurgical complications, either infectious or medically related, which could result in the loss of zygomatic implants.

The cumulative failure rates of zygomatic implants and conventional implants were 1.6% and 5.2%, respectively, with follow-up time periods of 6 months to 12 years [12]. Reports on classic surgical approaches have shown that the survival rate of zygomatic implants is higher than that of conventional implants [21]. Ahlgren et al. (2006) reported a 100% success rate for zygomatic implants achieved over a follow-up period of 11 to 49 months [22].

In our study, the survival rate of zygomatic implants was 99.0%, while for the standard implants, it was 99.5% over a follow-up of 120 months (10 years). The results of this study are consistent with those described in the literature, revealing extremely high survival rates. However, a longer follow-up period is presented, compared to the available literature.

### 4.2. Surgical Approach

The pooled incidence rates for the original surgical technique (OST) reveal an incidence of 9.53% for sinusitis, 7.5% for soft tissue infection, 10.78% for paresthesia, 4.58% for oroantral fistula formation, and 6.91% for surgical-related complications, alongside 56 reported cases of prosthesis-related issues [23]. In comparison, the pooled incidence rates for the anatomy-guided technique (AGA) include 4.39% for sinusitis, 4.35% for soft tissue infection, 0.55% for paresthesia, 1.71% for oroantral fistula formation, 1.6% for surgical-related complications, and 104 reported instances of prosthesis-related complications [23]. It should be noted, however, that these percentages may not fully encompass the scope of the complications associated with both techniques, as many clinical studies did not report the presence or absence of complications comprehensively.

In this systematic review, we aimed to evaluate and compare the survival and complication rates of zygomatic implants using both the OST and the anatomy-guided approach (AGA) in patients with severely atrophic maxillae. The results indicate that both techniques achieved a commendable implant survival rate with few reported complications. Overall, our findings suggest that there is a parity in clinical outcomes concerning implant survival between the two approaches. Specifically, the 923 zygomatic implants placed using the OST demonstrated a survival rate between 90.3% and 100%, while the 1302 implants placed via the anatomy-guided approach showed a survival rate ranging from 90.4% to 100% [23].

The intrasinus approach generated 1.41- and 4.27-fold higher stress at the bone–implant interface and the zygomatic implant body, respectively, under vertical loading than the extramaxillary approach [10]. However, the reverse was observed under lateral loading where the extramaxillary approach showed an increased stress level at the bone–implant interface by 2.48-fold [10]. The zygomatic implant body in the extramaxillary approach also exhibited micromotion, with a magnitude twice as high as that seen with the intrasinus approach under lateral loading. Both techniques may be used for the treatment of severely atrophic maxillae; however, the intrasinus approach is more favorable if lateral loading is a major concern [10].

In this research, the zygomatic implants were placed according to the principles outlined by the ZAGA classification [14]. In the first quadrant, ZAGA I was applied in 80% of cases, followed by ZAGA II in 12% and ZAGA 0 in 6% of cases. In the second quadrant, ZAGA I was applied in 74% of cases, followed by ZAGA II in 16% and ZAGA 0 in 10% of cases. Zygomatic implants were placed according to the lateral maxillary sinus concavity wall as well as the palatal resorption of the maxillary alveolus. It is worth highlighting, however, the paramount importance of quad-cortical stabilization in all clinical cases, as this has proven to be the crucial factor for the results obtained. This surgical principle, although described in several publications, is not implemented as clearly in any of them.

### 4.3. Loading Protocol

The primary advantage of graftless techniques is the ability to achieve immediate loading, enabling the prompt restoration of both function and esthetics post-surgery. The analysis revealed a significant difference in the prevalence of delayed versus immediate loading protocols among the various approaches: 77.7% for the Osteotome Sinus Technique (OST) and 22.3% for the immediate protocols, in contrast with the anatomy-guided technique, which showed 10.4% for delayed and 89.6% for immediate loading. This disparity may reflect the impact of recent advancements in anatomical-guided techniques and improvements in implant materials. The failure rates of the immediate loading protocols were recorded at 2.56% for the OST and 1.75% for the anatomy-guided technique. A comprehensive review involving 4566 zygomatic implants identified a failure rate of 1.7% for the immediate loading protocol based on the analysis of 103 failures [23].

The clinical course of the delayed zygomatic cases suggests a low-grade infection [24,25]. These types of infections are caused by low-virulent bacteria, such as Staphylococcus epidermidis or Propionibacterium acnes, which can infect at a later stage and manifest the associated complications even years after the original surgery [26]. For this reason, Ottenhausen et al. (2018) recommend a routine bacterial culture and a 16S ribosomal ribonucleic acid (rRNA) polymerase chain reaction (PCR) to identify these bacteria [26].

In this study, 80% of the cases received immediate loading (within 24 to 48 h after implant insertion), 14% received a delayed loading (more than 4 months after implant insertion), and 6% received early loading (between 48 h and 3 months). In most clinical cases included in this study, immediate function was performed, which is in line with the most recently published literature. However, contrary to some studies, namely Vrielinck et al.’s and Lopes et al.’s studies, there was no increase in the rate of associated complications [24,25].

### 4.4. Microbiology

A successful dental implant relies on optimal hard and soft tissue conditions. This includes appropriate dimensions (vertical, sagittal, and transverse) and bone quality, together with healthy soft tissues around the implant shoulder, including an area of non-mobile keratinized gingiva. The only widely accepted distinctive design in implant dentistry is the long zygomatic implant [27].

In a study by Lombardo et al. (2016), the microbiological results were correlated with a history of periodontitis. The periodontal patients, in fact, showed a higher presence of periodontal pathogens compared to the non-periodontal group (53.8% vs. 28.6%), and three patients with a history of periodontitis also exhibited a higher bacterial load of red complex pathogens. P. gingivalis was found more frequently (46.2%), especially in periodontal patients. Moreover, P. gingivalis was noted in all the periodontal patients who had the highest loads of periodontal pathogen [28]. The patient’s history of periodontitis also appeared to be relevant to bacterial colonization surrounding the zygomatic implants [28,29].

In all patients included in this study, the keratinized mucosa around the zygomatic and standard implants was carefully evaluated. This assessment was carried out intraoperatively with particular care in suturing, as well as during the final impressions phase. In the absence of keratinized mucosa, a free gingival graft would be performed before the placement of the final fixed implant-supported prosthesis.

### 4.5. Bone Resorption

Publications from the early 1990s defined success criteria for osseointegration and reported an acceptable bone loss of 1 mm in the first year, followed by an annual bone loss of 0.1 mm or 0.2 mm. Currently, reports of criteria do not exist. Consequently, the reported cumulative bone level changes show no evidence for a pathological process and should be considered acceptable [30,31]. Implant success was assessed according to several clinical and radiographic parameters, namely absence of pain; absence of progressive marginal bone resorption or peri-implant radiolucency; and absence of excessive bleeding during peri-implant probing or implant mobility [30,31].

A key factor in dental implant success or failure depends on the transmission of stress to the surrounding bone. Inappropriate loading may result in the concentration of stress in the bone around the implant, which could lead to bone resorption. It is known that the vertical load component plays a major role in masticatory loading. Conversely, the role of the horizontal load component should not be overlooked, although its impact is minimal, especially when an angled implant is used [13,32].

In our study, no significant statistical differences were found between T0, T1, and T2 concerning zygomatic bone area (first and second quadrants), the upper contact distance between the zygomatic bone and the zygomatic implant (first and second quadrants), and the lower contact distance between the zygomatic bone and the zygomatic implant (first and second quadrants). The bone resorption values described in the literature are based on standard implants and not zygomatic implants. Therefore, it becomes difficult to compare the results obtained in this study with other similar studies.

### 4.6. Bone Density

Type 4 bone quality is often found in the posterior maxilla, and the poor quality of bone in this region has been considered a factor contributing to increased implant failure [33]. The zygomatic implant offers anchorage for a fixed bridge, resorting to less invasive surgery compared to bone augmentation procedures [34]. The combination of zygomatic implants with anterior standard implants may positively impact implant stability during bone healing with immediate occlusal loading prostheses [31,35]. Rehabilitation with zygomatic implants requires sufficient bone volume in the anterior maxilla, with a minimum height of 10 mm and width of 4 mm, to allow for the placement of two to four conventional implants [36]. If the volume of bone in the anterior region is insufficient, then ideal conditions for bone grafting and guided bone regeneration must be provided [36].

Both clinical experience and theoretical modeling suggest that effective axial loading of the zygomatic implant is achieved through cross-arch stabilization with a rigid splint framework using at least four implants with adequate anterior–posterior spread [37]. Following specific prosthetic, biomechanical, and anatomical factors, establishing the entrance point depends on the vertical and horizontal resorption of the alveolar/basal process and the curvature of the anterior maxillary wall [23].

From a prosthodontic perspective, the starting point (implant head emergence) should be at or close to the top of the alveolar ridge crest. When the residual bone at the sinus floor level has adequate thickness and width (minimum: 4 mm height, 6 mm width) in a patient without a history of periodontitis, the position of the entry point should be near the middle portion of the crest, with an intrasinus starting path for the implant if the maxillary wall is flat or convex [23]. When the crestal bone height or thickness is inadequate, the alveolar entrance point should be shifted to the buccal, regardless of the maxillary wall curvature [23]. Based on the maxillary wall concavity and the height of the new bone, the osteotomy is shaped like a tunnel or canal [23].

In this research, highly significant statistical differences at an alpha level of 0.01 were identified between T0, T1, and T2 concerning zygomatic bone density, both in the first and in the second quadrants. The post hoc Bonferroni test revealed that significant statistical differences occurred between T0 and the remaining timepoints (T1 and T2), with the latter two exhibiting similar values among them. Bone density values described in the literature are based on standard implants and not on zygomatic implants. Therefore, it becomes difficult to compare the results obtained in this study with similar studies.

### 4.7. Complications

In a study conducted by Brennand et al. [18] it was observed that the overall prevalence of sinusitis was 14.2%, with an average follow-up period of 65.4 months. The approach to implant placement—whether intrasinus or extra-sinus—was found to be influenced by the patient’s unique anatomy and the desired prosthetic envelope [18]. When sinusitis was identified, treatment with antibiotics and/or a surgical meatotomy was generally successful, with no notable adverse consequences reported. While instances of zygomatic implant failure due to loss of osseointegration were infrequent in the context of sinusitis, it appears that surgical removal was often the preferred response to address unresolved sinusitis.

In this study, 8% of complications were recorded, present in four clinical cases, among which the following stand out: loss of a standard implant in anatomical position 2.1, two cases of postop sinusitis, and a fracture of a second quadrant zygomatic implant. The complication rates in this study were lower than those described in the literature; this fact could be explained by the fact that all surgical interventions were performed in a surgical center under general anesthesia, which allowed for a higher degree of asepsis and disinfection.

### 4.8. Prosthesis Survival

The prosthesis survival rates have been observed to range from 82% to 100%, with a mean survival rate of 94% at 76 months of follow-up. This favorable outcome aligns closely with the survival rates of zygomatic implants. It is important to consider that early failures of zygomatic implants may have influenced the survival of provisional prostheses, although this is unlikely to have impacted the definitive reconstructions [18]. When examining reconstructions supported solely by conventional implants, no statistically significant differences were noted in the materials used for full-arch screw-retained prostheses [38]. The prevalence of acrylic/acrylic teeth in many studies likely played a role in achieving these positive survival rates, as this design allows for repair rather than complete replacement. Complications such as chipping of the veneering resin and loss of retention have been reported, with the former being notably common [38].

Furthermore, instances of screw and abutment loosening have been reported frequently [39,40]. While these complications exist in the existing literature, it is possible that the unique dynamics of zygomatic reconstructions, including increased movement of the prosthesis due to bending forces, may contribute to the incidence rates [39,40,41]. These technical challenges were observed regardless of whether zygomatic implants were splinted to conventional implants or whether the prosthesis was solely supported by four zygomatic implants. Lastly, it is worth considering that such bending phenomena could also lead to fractures of the veneering material detached from the metal substructure [18].

In this study, the final fixed implant-supported prosthesis survival rate was 100.0%, over a follow-up of 120.0 months (10 years). All the prosthesis placed were screw-retained, allowing for reversibility for control and maintenance. The values obtained in this study align with those described in the literature, revealing extremely high survival rates; however, a longer follow-up period is presented compared to the available literature.

## 5. Limitations

As limitations to this study, we could point out the sample size of 50 patients, which may not be very large. Additionally, the placement of implants in the premaxilla area may not always be identical across all cases, due to differences in bone availability. Similarly, the length of the zygomatic implants does not always maintain a correlation between both zygomas. We can also highlight the absence of studies similar to ours, meaning that both the methodology and the results of this study cannot be compared.

## 6. Conclusions

The objective of this research was to retrospectively analyze ten years of rehabilitation with zygomatic implants, focusing on whether there are losses at the level of the zygoma, proposing as a null hypothesis that these implants remain stable over time without bone resorption at the zygomatic bone level.

The evaluation of resorption at the level of the zygoma, after ten years of zygomatic implant placement, reveals no significant losses between the initial and final controls. Therefore, it follows that this type of implant rehabilitation represents a viable alternative approach in patients with bone atrophy of the maxilla, offering a predictable therapeutic solution that enables immediate full function and excellent long-term success rates.

## Figures and Tables

**Table 1:** Sociodemographic and general clinical characterization of the sample.

Variable	Statistical Parameter
Sample *N*	50
Age *Mean ± SD, years*	60.32 ± 10.43
Gender *n (percentage)*	Male: 17 (34.0); Female: 33 (66.0).
Zygoma Anatomy-Guided Approach (ZAGA) (1st Quadrant) *n (percentage)*	ZAGA 0: 3 (6.0); ZAGA I: 40 (80.0); ZAGA II: 6 (12.0); ZAGA III: 1 (2.0); ZAGA IV: 0 (0.0).
Zygoma Anatomy-Guided Approach (ZAGA) (2nd Quadrant) *n (percentage)*	ZAGA 0: 5 (10.0); ZAGA I: 37 (74.0); ZAGA II: 8 (16.0); ZAGA III: 0 (0.0); ZAGA IV: 0 (0.0).
Zygomatic Implant Length (1st Quadrant) *n (percentage)*	4.5 × 32.5 mm: 9 (18.0); 4.5 × 35 mm: 12 (24.0); 4.5 × 37.5 mm: 9 (18.0); 4.5 × 40 mm: 9 (18.0); 4.5 × 42.5 mm: 5 (10.0); 4.5 × 45 mm: 4 (8.0); 4.5 × 47.5 mm: 1 (2.0); 4.5 × 50 mm: 1 (2.0).
Zygomatic Implant Length (2nd Quadrant) *n (percentage)*	4.5 × 32.5 mm: 7 (14.0); 4.5 × 35 mm: 10 (20.0); 4.5 × 37.5 mm: 14 (28.0); 4.5 × 40 mm: 9 (18.0); 4.5 × 42.5 mm: 6 (12.0); 4.5 × 45 mm: 2 (4.0); 4.5 × 47.5 mm: 1 (2.0); 4.5 × 50 mm: 1 (2.0).
Zygomatic Implant Torque (1st Quadrant) *Mean ± SD, Newtons*	48.70 ± 11.19
Zygomatic Implant Torque (2nd Quadrant) *Mean ± SD, Newtons*	49.70 ± 10.95
Loading Protocol *n (percentage)*	Immediate: 40 (80.0); Early: 3 (6.0); Delayed: 7 (14.0).
Antagonist Occlusion *n (percentage)*	Natural Teeth: 15 (30.0); Removable Partial Denture: 1 (2.0); Overdenture: 4 (8.0); Partial Fixed Implants: 4 (8.0); Total Fixed Implants: 26 (52.0).
Complications *n (percentage)*	No: 46 (92.0); Yes: 4 (8.0).

**Table 2:** Specific clinical characterization of the sample concerning the implants placed.

Variable	Statistical Parameter
Standard Implant 1 Position *n (percentage)*	1.1 position: 27 (54.0); 1.2 position: 15 (30.0); 1.3 position: 5 (10.0); 1.4 position: 1 (2.0); 1.7 position: 2 (4.0).
Standard Implant 1 Length *n (percentage)*	3.25 × 8.5 mm: 5 (10.0); 3.25 × 10 mm: 4 (8.0); 3.75 × 7 mm: 1 (2.0); 3.75 × 8.5 mm: 7 (14.0); 3.75 × 10 mm: 12 (24.0); 3.75 × 11.5 mm: 2 (4.0); 3.75 × 15 mm: 3 (6.0); 4.1 × 7 mm: 8 (16.0); 4.1 × 8.5 mm: 2 (4.0); 4.1 × 10 mm: 2 (4.0); 4.5 × 8.5 mm: 1 (2.0); 4.5 × 10 mm: 2 (4.0); 4.5 × 15 mm: 1 (2.0).
Standard Implant 1 Torque *Mean ± SD, Newtons*	43.10 ± 11.51
Standard Implant 2 Position *n (percentage)*	1.2 position: 5 (10.0); 1.3 position: 18 (36.0); 1.4 position: 6 (12.0); 1.6 position: 3 (6.0); 1.7 position: 1 (2.0); 2.1 position: 8 (16.0); 2.2 position: 6 (12.0); 2.3 position: 3 (6.0).
Standard Implant 2 Length *n (percentage)*	3.25 × 8.5 mm: 4 (8.0); 3.25 × 10 mm: 2 (4.0); 3.75 × 7 mm: 2 (4.0); 3.75 × 8.5 mm: 8 (16.0); 3.75 × 10 mm: 15 (30.0); 3.75 × 11.5 mm: 3 (6.0); 3.75 × 13 mm: 1 (2.0); 3.75 × 15 mm: 2 (4.0); 4.1 × 7 mm: 4 (8.0); 4.1 × 8.5 mm: 2 (4.0); 4.1 × 10 mm: 3 (6.0); 4.1 × 11.5 mm: 1 (2.0); 4.1 × 13 mm: 1 (2.0); 4.5 × 13 mm: 1 (2.0); 5 × 10 mm: 1 (2.0).
Standard Implant 2 Torque *Mean ± SD, Newtons*	40.30 ± 13.49
Standard Implant 3 Position *n (percentage)*	1.6 position: 1 (2.0); 2.1 position: 19 (38.0); 2.2 position: 11 (22.0); 2.3 position: 2 (4.0); 2.4 position: 3 (6.0); 2.7 position: 2 (4.0).
Standard Implant 3 Length *n (percentage)*	3.25 × 8.5 mm: 2 (4.0); 3.25 × 10 mm: 3 (6.0); 3.75 × 7 mm: 4 (8.0); 3.75 × 8.5 mm: 7 (14.0); 3.75 × 10 mm: 5 (10.0); 3.75 × 11.5 mm: 3 (6.0); 3.75 × 15 mm: 3 (6.0); 4.1 × 7 mm: 2 (4.0); 4.1 × 8.5 mm: 1 (2.0); 4.1 × 10 mm: 1 (2.0); 4.5 × 8.5 mm: 3 (6.0); 4.5 × 10 mm: 3 (6.0); 5 × 11.5 mm: 1 (2.0).
Standard Implant 3 Torque *Mean ± SD, Newtons*	43.68 ± 14.17
Standard Implant 4 Position *n (percentage)*	2.2 position: 4 (8.0); 2.3 position: 14 (28.0); 2.4 position: 6 (12.0); 2.6 position: 4 (8.0).
Standard Implant 4 Length *n (percentage)*	3.25 × 8.5 mm: 2 (4.0); 3.75 × 7 mm: 1 (2.0); 3.75 × 8.5 mm: 6 (12.0); 3.75 × 10 mm: 11 (22.0); 3.75 × 11.5 mm: 1 (2.0); 4.1 × 7 mm: 1 (2.0); 4.1 × 8.5 mm: 1 (2.0); 4.1 × 11.5 mm: 1 (2.0); 4.1 × 15 mm: 1 (2.0); 4.5 × 8.5 mm: 1 (2.0); 4.5 × 10 mm: 1 (2.0); 5.0 × 13 mm: 1 (2.0).
Standard Implant 4 Torque *Mean ± SD, Newtons*	37.68 ± 16.70

**Table 3:** Patients’ follow-ups.

Variable	Statistical Parameter
Zygomatic Bone Area (1st Quadrant) (mm^2^) *Mean ± SD, Newtons*	T0: 205.96 ± 63.54; T1: 205.96 ± 63.54; T2: 205.96 ± 63.54.
Zygomatic Bone Area (2nd Quadrant) (mm^2^) *Mean ± SD, Newtons*	T0: 204.18 ± 64.91; T1: 204.18 ± 64.91; T2: 204.18 ± 64.91.
Zygomatic Bone Density (1st Quadrant) (HU) *Mean ± SD, Newtons*	T0: 1077.23 ± 264.90; T1: 2642.06 ± 550.33; T2: 2853.04 ± 530.26.
Zygomatic Bone Density (2nd Quadrant) (HU) *Mean ± SD, Newtons*	T0: 1030.46 ± 314.13; T1: 2457.86 ± 561.85; T2: 2696.60 ± 583.29.
Upper Contact Distance between Zygomatic Bone and Zygomatic Implant (1st Quadrant) (mm) *Mean ± SD, Newtons*	T0: No valid cases; T1: 9.01 ± 3.19; T2: 9.10 ± 3.23.
Upper Contact Distance between Zygomatic Bone and Zygomatic Implant (2nd Quadrant) (mm) *Mean ± SD, Newtons*	T0: No valid cases; T1: 8.04 ± 3.02; T2: 8.20 ± 3.19.
Lower Contact Distance between Zygomatic Bone and Zygomatic Implant (1st Quadrant) (mm) *Mean ± SD, Newtons*	T0: No valid cases; T1: 13.70 ± 4.74; T2: 13.61 ± 4.62.
Lower Contact Distance between Zygomatic Bone and Zygomatic Implant (2nd Quadrant) (mm) *Mean ± SD, Newtons*	T0: No valid cases; T1: 14.69 ± 4.96; T2: 14.65 ± 4.98.

**Table 4:** Repeated-measures ANOVA.

Variable	Test Statistics (F)	*p*-Value
Zygomatic Bone Area (1st Quadrant) (mm^2^) *Mean ± SD. Newtons*	0.000	1.000
Zygomatic Bone Area (2nd Quadrant) (mm^2^) *Mean ± SD. Newtons*	0.000	1.000
Zygomatic Bone Density (1st Quadrant) (HU) *Mean ± SD. Newtons*	215.782	<0.001 ***
Zygomatic Bone Density (2nd Quadrant) (HU) *Mean ± SD. Newtons*	161.364	<0.001 ***
Upper Contact Distance between Zygomatic Bone and Zygomatic Implant (1st Quadrant) (mm) *Mean ± SD. Newtons*	0.021	0.886
Upper Contact Distance between Zygomatic Bone and Zygomatic Implant (2nd Quadrant) (mm) *Mean ± SD. Newtons*	0.065	0.800
Lower Contact Distance between Zygomatic Bone and Zygomatic Implant (1st Quadrant) (mm) *Mean ± SD. Newtons*	0.010	0.922
Lower Contact Distance between Zygomatic Bone and Zygomatic Implant (2nd Quadrant) (mm) *Mean ± SD. Newtons*	0.001	0.974

**Table 5:** Post hoc Bonferroni inferential test.

Variable	Comparison		Mean Difference (I–J)	Std. Error	*p*-Value
Zygomatic Bone Density (1st Quadrant) (HU)	T0	T1	−1564.820 *	93.396	<0.001
		T2	−1775.800 *	93.396	<0.001
	T1	T0	1564.820 *	93.396	<0.001
		T2	−210.980	93.396	0.076
	T2	T0	1775.800 *	93.396	<0.001
		T1	210.980	93.396	0.076
Zygomatic Bone Density (2nd Quadrant) (HU)	T0	T1	−1427.400 *	100.305	<0.001
		T2	−1666.140 *	100.305	<0.001
	T1	T0	1427.400 *	100.305	<0.001
		T2	−238.740	100.305	0.056
	T2	T0	1666.140 *	100.305	<0.001
		T1	238.740	100.305	0.056

## Data Availability

All data is contained in the presented article.
